# Case Report: Modified Latissimus Dorsi Muscle Transfer for Radial Palsy, Presentation of a New Technique

**Published:** 2008-12-23

**Authors:** Shahram Nazerani, Mohammad Hosein Kalantar Motamedi

**Affiliations:** ^a^Department of Surgery, Iran University of Medical Sciences, Tehran, Iran; and; ^b^Trauma Research Center, Baqiyatallah Medical Sciences University, and Attending Faculty, Azad University of Medical Sciences, Tehran, Iran

## Abstract

**Objective:** The latissimus dorsi muscle transfer finger and wrist extension is a well-known procedure for patients needing replantation or in brachial plexus injuries. To increase the length of the transferred muscle to reach to the finger and wrist extensors, the other authors suggest extending the muscle length by incorporating the iliac crest fascia, which not only prolongs the operation time but also minimizes the chances of a healthy and viable muscle-tendon junction. We present a modification of the standard latissimus dorsi transfer whereby the whole muscle (not partial harvest) is transferred and extended by tendon graft to minimize the distal muscle-tendon problems and inefficient muscle excursion commonly encountered with the thus far reported techniques. **Methods:** In a 12-year period (1996–2008), 5 patients were treated. Guidelines for patient selection were (1) complete high radial nerve palsy with no simpler solution to address the problem, (2) supple joints and gliding tendons, (3) good patient motivation, and (4) free muscle transfer not feasible or risky. **Results:** Five male patients aged 18 to 45 were treated for the absence of wrist and finger extension due to radial nerve damage or extensor group destruction. They were able to use the transferred muscle for extension with minimal training and physiotherapy. **Conclusion:** (1) Transferring the whole muscle ensures a complete neural arborization and better excursion. (2) It ensures a safe tendon suture wherein the muscle-tendon unit is far from the distal end of the skin flap (less possibility of necrosis).

The latissimus dorsi (LD) is a triangular pennate muscle. In pennate muscles, the excursion length of muscles is much less than that in strap muscles. Thus, when a partial pennate muscle is harvested because the direction of muscle fibers is oblique, these fibers actually have no function for longitudinal contraction and act more like a tendon when transferred partially. Second, the vascular supply is also compromised such that the important area of “muscle-to-extensor tendon suture” is prone to necrosis and suture slackening (Fig 1).

The muscle-tendon junction is usually too bulky to be covered by the skin and amenable to exposure followed by infection. Owing to inability of adjusting the muscle tension at the first setting or suture loosening due to ischemia, inefficient muscle excursion is another observed and common problem in the partial harvest of the muscle with or without iliac fascia incorporated. With these aforementioned problems facing us, we modified the muscle transfer technique and assessed our results with this new technique in 5 patients.

## METHODS

**Figure 2:**
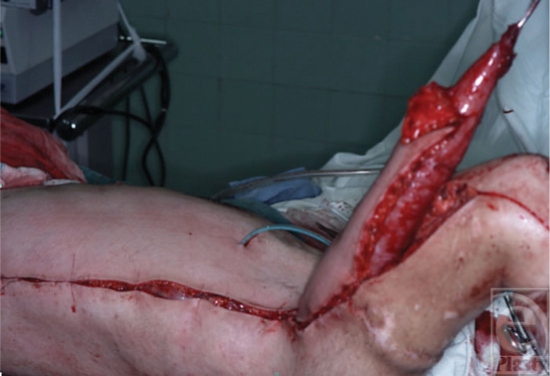
The complete muscle rolled and partially set in the gutter before the tendon graft attachment.

**Figure 3:**
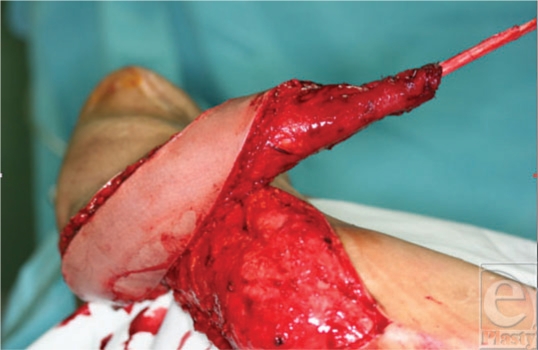
The attached tendon graft to the muscle with a very small muscle-tendon “knot.”

**Figure 4:**
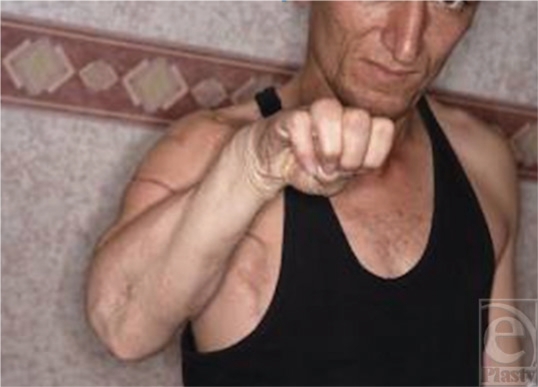
A complete through arm amputation. The finger flexion is seen with the latissimus dorsi muscle relaxed so that the patient can grasp.

**Figure 5:**
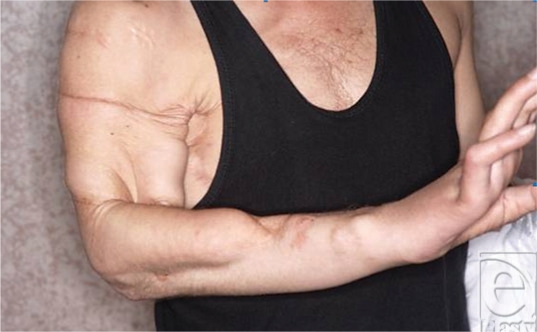
The extension of wrist and fingers is observed, near-complete extension of wrist and fingers is seen, and even some hyperextension of the metacarpophalangeal joint due to the absence of interossei and lumbricales muscles is noted.

The LD muscle is completely dissected free along with an oblong skin paddle. The insertion is severed from the humerus (bipolar transfer), the muscle is rolled and sutured to itself, and the muscle with the paddle of skin is transferred in a gutter running from the lateral upper arm to the upper dorsal forearm (Fig 2). The tensor fasciae latae (TFL) tendon graft is harvested, a slit is made in the tendon graft, and then it is passed through a “slit” in the distal part of the transferred and rolled muscle. The slit in the muscle will have no effect on its blood supply because the muscle is split and not cut (Fig 3). The tendon is passed through the slit made at one of its end such that it makes a loop around the muscle, and a strong and, at the same time, a small tendon-muscle joining is created. The graft is then passed through a mid-forearm tunnel and sutured to the extensors prepared in a separate incision in the distal forearm. The distal suture to the extensors is easily made by this method. Because the tendon firmly grasps the muscle, proper tension can be adjusted. The forearm skin covers the distal suture line. The average tendon graft length is 17 cm, but all the TFL length should be harvested and later it could be tailored to the best tension. This is possible because the tendon graft-LD muscle junction is not sutured but is a woven junction so that it can accept much more tension. The arm is held in elbow flexion and the wrist and fingers in extension for a period of 6 to 8 weeks (Figs 4 and 5).

## RESULTS

The patients were evaluated 1 and 3 years after the operation; 2 of them had returned to their previous jobs, 1 as a tractor driver, and the other 3 had retired owing to the severity of injuries.

All the patients were satisfied with their ability to extend the wrist and fingers. They were able to perform their daily chores, and when questioned, they were satisfied with their operation.

Owing to the suturing of wrist and finger extensors to 1 muscle, separate wrist and finger extension was not possible.

The strength of the transferred muscle was evaluated. It ranged from a low of 2.5 to 8 kg, with a median of 6 kg, which is 4 kg higher than the standard bipolar method reported in the literature. The range of motion of the fingers in extension was observed. The total active motion ranged from 155 to 170, with a mean of 160 (normal value = 205).

**Figure 6:**
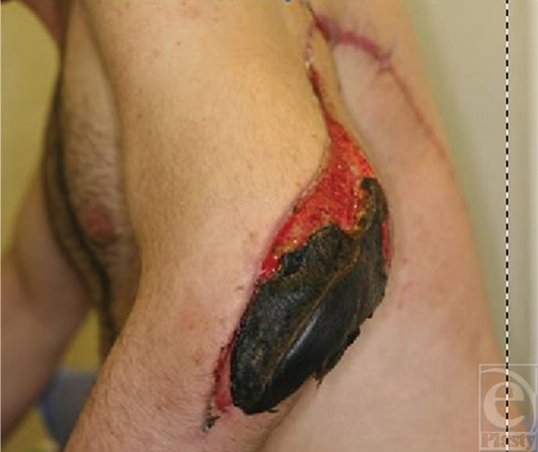
Distal flap skin necrosis, with tendon graft survived, an advantage of this technique, which in standard procedure, the muscle-tendon junction otherwise would have been exposed.

**Figure 7:**
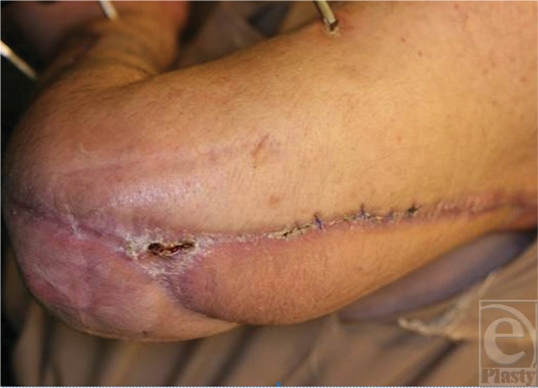
The wound healed with no damage to the tendon graft.

**Figure 8:**
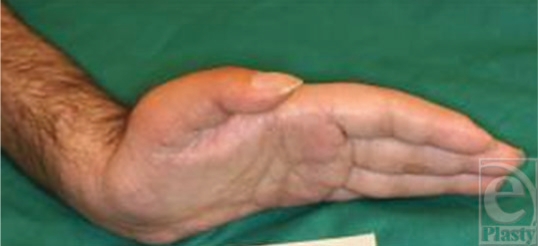
Extension is observed, with near-complete wrist extension.

**Figure 9:**
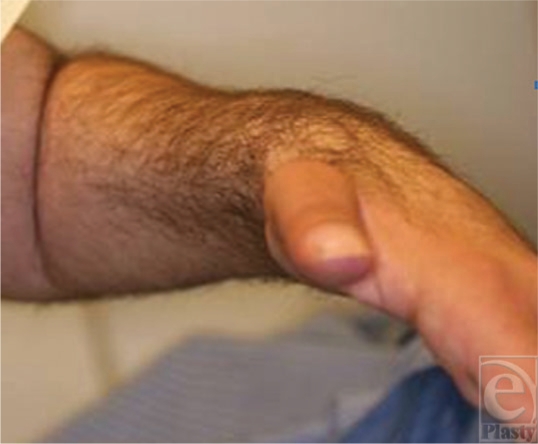
Owing to some tenodesis effect, the complete wrist flexion is not possible.

Distal flap skin necrosis was seen in 1 patient. Because the tendon-muscle junction was more proximal, it was not exposed after removing the necrotic skin, an advantage of this technique, which in the standard procedure, the muscle-tendon junction otherwise would have been exposed (Fig 6). The wound healed by secondary intention (Fig 7). Extension was observed, with near-complete wrist extension (Figs 8 and 9).

### Case presentation

**Figure 10:**
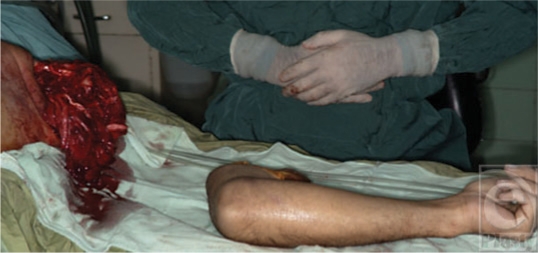
The amputated avulsed upper extremity. The radial nerve had been avulsed completely from the plexus and could not be coaptated.

**Figure 11:**
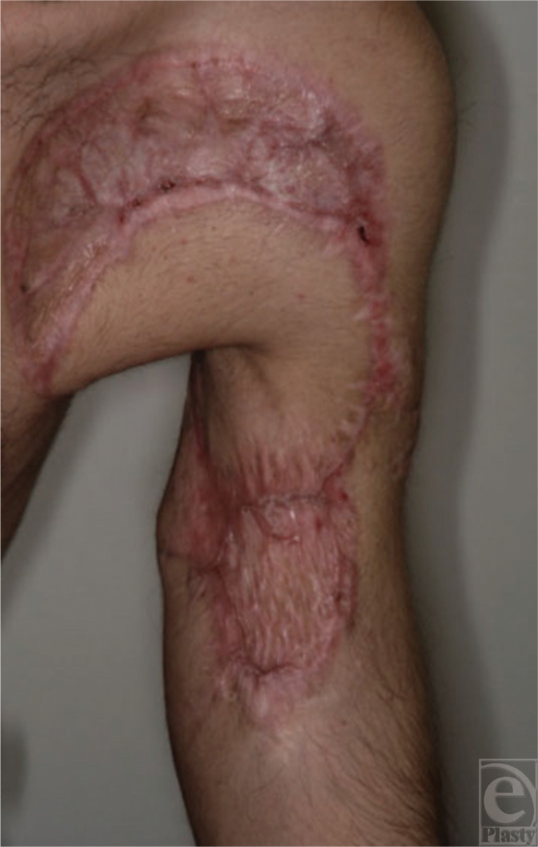
The skin grafts for the exposed wounds are done and the replant has healed completely.

**Figure 12:**
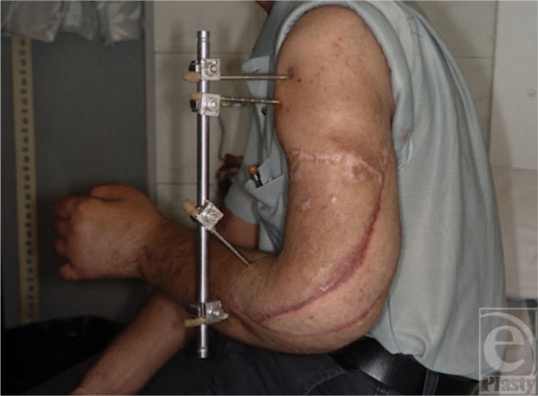
The healed latissimus dorsi muscle and the external fixator holding the elbow in flexion.

**Figure 13:**
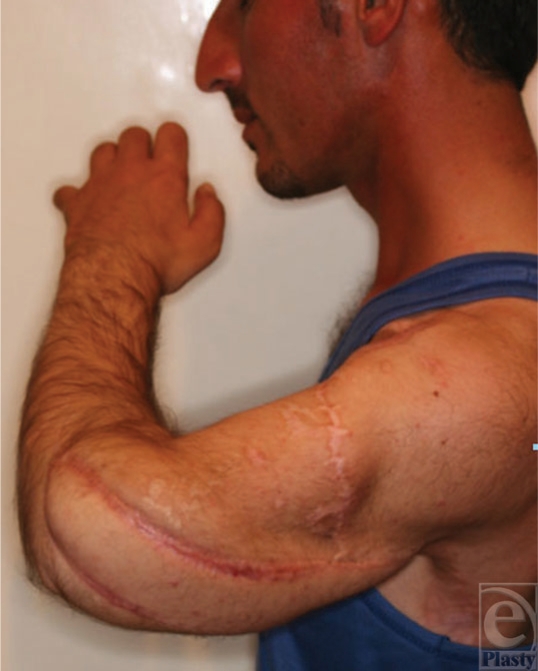
The flexed fingers and the good wrist neutral position.

**Figure 14:**
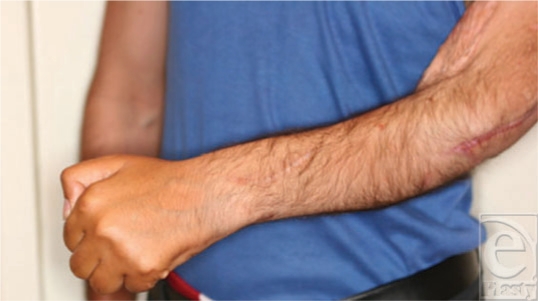
Wrist extension.

A 28-year-old man had his arm caught in farm machinery, which amputated the extremity at the upper arm (Fig 10). The patient was admitted to our hospital 6 hours after injury. After resuscitation, the replantation was begun. All the nerves except the radial nerve, which had been avulsed and was unrepairable, were coaptated. The follow-up period was uneventful. The exposed wounds (after 3 weeks) were skin grafted (Fig 11). Nine months later, the nerve recovery was seen in the median and ulnar nerve areas. After 16 months, a pedicled LD musculocutaneous flap was designed and transferred to the forearm and was lengthened by a TFL graft and sutured to extensors (Fig 12). The postoperative period was uneventful. The patient began to extend his wrist and fingers 6 weeks after the removal of the external fixator (Figs 13 and 14).

## SUMMARY

The LD muscle transfer of finger and wrist extension is a well-known procedure in patients in whom the transfer of median and ulnar area muscles is not possible. Berger and Brenner^1^ showed that free neurovascular LD transfer developed a maximal muscular strength of 2 to 4 kg in the unipolar variation and 1 to 2 kg for the bipolar LD. They also showed good results after secondary replacement operations for the reconstruction of the elbow joint after lesion of the brachial plexus.^2^ Elbow flexion was reconstructed by bipolar LD transfer in 35 patients.^2^

Gousheh et al^3^ described the technique of transferring the LD muscle as an island flap for the restoration of digital flexion or extension in 28 patients. The LD muscle is raised down to the posterior iliac crest and prolonged with the glutealsuperficial fascia. They believe that this method is particularly suitable for extensive and prolonged paralysis of the lower elements of brachial plexus and can be used also for severe Volkmann's contracture.^3^

Doi et al^4^ stated that functional LD island pedicle musculocutaneous flaps can be used to restore the flexion or extension of the wrist and digits. By retaining the facial origin of the LD from the posterior crest of the ilium, the entire muscle may be transferred without dividing its neurovascular pedicle and microneurovascular anastomoses. Its facial origin successfully reached the finger flexor or extensor muscles of the forearm. The transfers restored active finger flexion or extension.^4^ Favero et al^5^ stated that the transfer of functioning free muscle for the restoration of finger flexion is an uncommon procedure, and LD musculocutaneous free tissue transfer was performed in patients in an attempt to provide soft tissue coverage and active digital flexion. Although preoperative grip strength was doubled, postoperative strength was still only about 31% of that on the opposite side. All patients required at least 1 tenomyolysis or revision tenorrhaphy before the best clinical outcome was achieved.^5^ Chang et al^6^ ascribed upper extremity reconstruction to be very challenging owing to the unavailability of expendable local muscles.

We have transferred the complete LD muscle with a suitable skin paddle to the dorsal forearm through a gutter at the lateral border of the arm in 5 patients. The transfer is bipolar and muscle insertion is sutured to the dorsal upper arm muscles and deep fascia. The TFL tendon is harvested and passed through a slit in the muscle and fixed to the extensors of the wrist and fingers at a separate incision. All the previous authors have tried to lengthen the LD muscle by gluteal fascia, but we think adding a tendon graft between the LD muscle and the extensors is stronger because proper tension can be applied to the muscle without fear of ischemia to the muscle and suture disruption at the extensor-muscle junction. An external fixator holds the elbow in flexion and the wrist and fingers in extension. A literature review revealed no similar technique. Total muscle transfer with added tendon graft has far superior activity than the partial muscle transfer sutured directly to the extensors. The results have been uniformly good, with less revisional reefing of muscle and satisfactory extensor action on joints, and a good range of motion is obtained.

The benefits of the modification, which we have added to LD transfer, are as follows: (1) transferring the whole muscle ensures a complete neural arborization to the end of the muscle, hence a better excursion than a partial muscle harvest. (2) It ensures a safe tendon suture in which the muscle-tendon unit is far from the distal end of the skin flap where the possibility of necrosis is higher. A tendon graft is added between the muscle and the extensor tendon group so that the tendon graft, and not the muscle, will be joined to extensor tendons and a more secure and less bulky coaptation is ensured. A tendon graft added to the muscle makes a small knot and passes easily in a subcutaneous tunnel, with the tendon sutures to the extensors completely covered. (3) A gutter made for placing the muscle has a lesser chance for hematoma and necrosis than a closed tunnel. (4) The maximum efficient length of the composite muscle-tendon unit can be obtained without fear of muscle necrosis or vascular compromise. (5) No revision was required in this series. Muscle necrosis was not seen. Distal skin necrosis and muscle exposure were observed in 1 patient, which healed without any adverse effects on the outcome.

Owing to the large muscle mass and the skin flap, the aesthetic appearance is not ideal, but the skin flap can be removed at a later stage for a better aesthetic appearance.

## Figures and Tables

**Figure 1:**
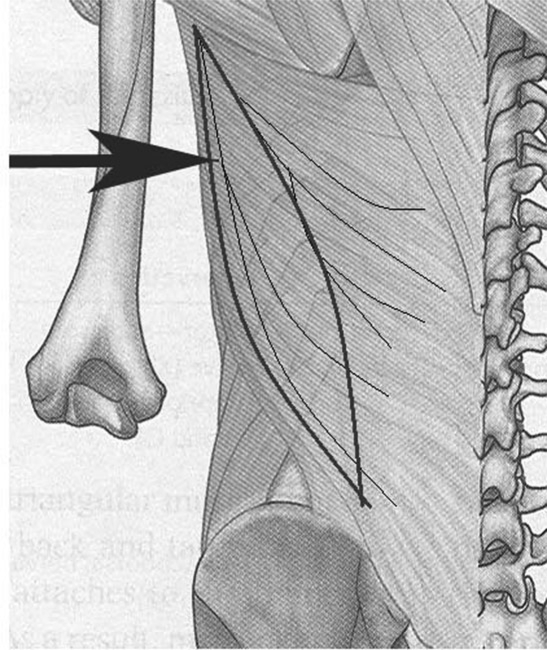
The schematic drawing of the latissimus dorsi muscle depicting that with partial transfer, the upper muscle fibers do not contribute to flexion.
